# Journey to the other side of the brain: asymmetry in patients with chronic mild or moderate traumatic brain injury

**DOI:** 10.2217/cnc-2022-0003

**Published:** 2023-01-31

**Authors:** David E Ross, John D Seabaugh, Jan M Seabaugh, Claudia Alvarez, Laura Peyton Ellis, Christopher Powell, Christopher Reese, Leah Cooper, Katherine Shepherd, for the Alzheimer's Disease Neuroimaging Initiative

**Affiliations:** 1 Virginia Institute of Neuropsychiatry, Midlothian, VA 23114, USA; 2 Neuroscience Department, Randolph Macon College, Ashland, VA 23005, USA; 3 Virginia Commonwealth University, Medical College of Virginia, Richmond, VA 23219, USA; 4 Neuroscience Department, University of North Carolina at Wilmington, Wilmington, NC 28403, USA; 5 Neuroscience Department, Virginia Polytechnic Institute & State University, Blacksburg, VA 24061, USA; 6 Neuroscience Department, James Madison University, Harrisonburg, VA 22807, USA

**Keywords:** asymmetry, MRI, NeuroGage^®^, NeuroQuant^®^, traumatic brain injury (TBI), volumetry

## Abstract

**Aim:**

Patients with chronic mild or moderate traumatic brain injury have some regions of brain atrophy (including cerebral white matter) but even more regions of abnormal brain enlargement (including other cerebral regions).

**Hypothesis:**

Ipsilateral injury and atrophy cause the eventual development of contralateral compensatory hypertrophy.

**Materials & methods:**

50 patients with mild or moderate traumatic brain injury were compared to 80 normal controls (n = 80) with respect to MRI brain volume asymmetry. Asymmetry-based correlations were used to test the primary hypothesis.

**Results:**

The group of patients had multiple regions of abnormal asymmetry.

**Conclusion:**

The correlational analyses supported the conclusion that acute injury to ipsilateral cerebral white matter regions caused atrophy, leading eventually to abnormal enlargement of contralateral regions due to compensatory hypertrophy.

Decades of research have shown that patients with traumatic brain injury (TBI) have abnormal asymmetry of brain regions [1–3]. It is especially prominent in patients with obvious or severe unilateral brain damage on the day of injury. For example, if a patient with moderate or severe TBI had a left frontal cerebral contusion on the day of injury, it would be common to see at 1 year follow-up frontal asymmetry characterized by left frontal encephalomalacia and more normal-appearing right frontal lobe, that is, abnormal asymmetry (L<R). Herein, the phrase ‘abnormal asymmetry (L<R)’ indicates “abnormal asymmetry with the left-sided volume being smaller than expected based on the size of the right”. This terminology does not necessarily indicate that the left-sided volume is abnormally small or that the right-sided region is abnormally large; it simply indicates that the left side is smaller than would be expected based on the size of the right.

A limitation of this area of research is that most of it was based on patients with severe TBI. Much less is known about abnormal asymmetry in patients suffering from chronic effects of mild or moderate TBI [4–6] (herein referred to as “patients with chronic mild or moderate TBI”), a group that generally might be expected to have less severe brain pathology than groups with severe TBI. So it remains unclear whether or to what extent patients with chronic mild or moderate TBI have abnormal asymmetry.

Some general discussion of brain volume asymmetry in TBI may be helpful to the reader. Is it better to measure brain volume asymmetry or to more simply and straightforwardly measure brain volume? In other words, why is measuring asymmetry helpful if we already have volume measurements that directly indicate whether a given brain region is abnormally small or abnormally large? (Note that herein, we will use the term “volume” to refer to simple, straightforward volume measurement, in other words, not asymmetry measurement; and we will use the term ‘asymmetry’ to refer to brain volume asymmetry; for a detailed description of our methods for calculating asymmetry, see the Methods/Brain Imaging section below). In many cases, asymmetry can reveal abnormalities when cross-sectional volume measurements are normal. Asymmetry measurements take advantage of the fact that normal people have brains that, to a first approximation, are bilaterally symmetric (note that with precise measurement, brains are not perfectly bilaterally symmetric; but for an initial understanding of brain asymmetry, this is a useful idea). Thus, if the volume of a given brain region was much smaller or larger than would be expected based on the volume of its contralateral counterpart, we would suspect that some abnormal volume change previously occurred.

For example, consider a case in which, prior to injury, a given patient had normal volume of the cerebral white matter (CWM) regions, with both regions at the 75th normative percentile; and these regions were bilaterally symmetrical, in other words, their asymmetry index was 0. Then imagine that after injury, the left CWM regions atrophied to the 25th normative percentile, which would be normal compared with a general normal control group, but which would be abnormally small in this patient compared with the pre-injury volume; in other words, there was longitudinal atrophy. Accordingly, if we measured asymmetry after injury, it would be abnormal, with the left-sided volume smaller than would be expected based on the size of the right. In this way, asymmetry can be considered to be analogous to longitudinal measurement.

However, asymmetry actually is cross-sectional measurement, not longitudinal. For example, imagine a similar case, except that we did not have volume data before injury (which is almost always the case in the real world). After injury, as with the previous example, the volumes would be normal and the asymmetry would be abnormal, with the left-sided volume smaller than would be expected based on the size of the right. Although there was abnormal asymmetry (L<R) after injury, because we could not measure volume before injury, we would not know whether the left-sided region atrophied, the right-sided region enlarged, or a combination of both. Furthermore, we would have no guarantee that there was any abnormally fast longitudinal volume change, even if we could have measured it; for example, the asymmetry could have been congenital. In summary, brain volume asymmetry can serve as a useful proxy for longitudinal measurement, but it is not a substitute.

Recent reports found that patients with chronic mild or moderate TBI had atrophy of CWM and a few other regions, but they had even more regions of abnormal enlargement, including cortical gray matter and subcortical regions [7–12]. The relatively new findings raised the question: what is the mechanism behind the enlargement? Previously we proposed two hypotheses: chronic neuroinflammation causes edema; and chronic neuronal dysfunction causes compensatory hypertrophy [11,12].

In support of the neuroinflammation hypothesis, studies in animals and humans have found that moderate to severe traumatic brain injury causes both acute and chronic neuroinflammation [13–20]. And neuroinflammation potentially can occur for years after TBI [15]. Therefore, it seems possible that chronic neuroinflammation and edema could cause the abnormally large brain volume in patients with chronic mild or moderate TBI.

Regarding the possibility of compensatory hypertrophy, enlargement could occur in less-injured regions connected to more-injured regions (Figure 1). It is well-known that when brain regions perform more tasks, they enlarge in volume; classic studies of this phenomenon include the keyboard player study [21] and the London taxi driver study [22].

**Figure 1.**
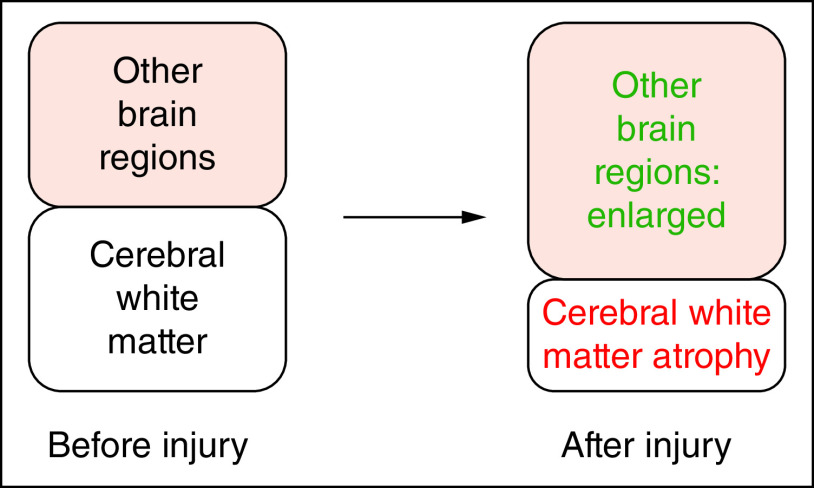
The hypothesis of compensatory hypertrophy posits that acute injury to cerebral white matter causes chronic atrophy of cerebral white matter and abnormal enlargement of less injured brain regions due to compensatory hypertrophy.

The hypotheses of neuroinflammation versus compensatory hypertrophy were explored in a recent report describing the case of a 42-year-old man who sustained a moderate traumatic brain injury characterized by day-of-injury left cerebral hemorrhage, followed 1.8 years later by CWM atrophy ipsilateral to the side of injury (i.e. left-sided) but contralateral (i.e., right) abnormal enlargement of the thalamus and multiple cerebral cortical gray matter regions [6]. (Herein, we will use the term ‘non-CWM regions’ to refer to regions other than CWM, which have been found to be abnormally large in patients with chronic mild or moderate TBI, including cerebral cortical gray matter and subcortical regions; see below for further information.) The presence of CWM atrophy and abnormal enlargement of non-CWM regions was typical of the pattern previously reported in a group study of patients with mild or moderate TBI [11]. Furthermore, the laterality of findings – ipsilateral CWM atrophy and asymmetry (L<R), combined with contralateral non-CWM abnormal enlargement and asymmetry (R>L) – suggested the hypothesis that acute ipsilateral injury led to chronic ipsilateral atrophy of CWM and contralateral compensatory hypertrophy of non-CWM regions (Figure 2, right side).

**Figure 2.**
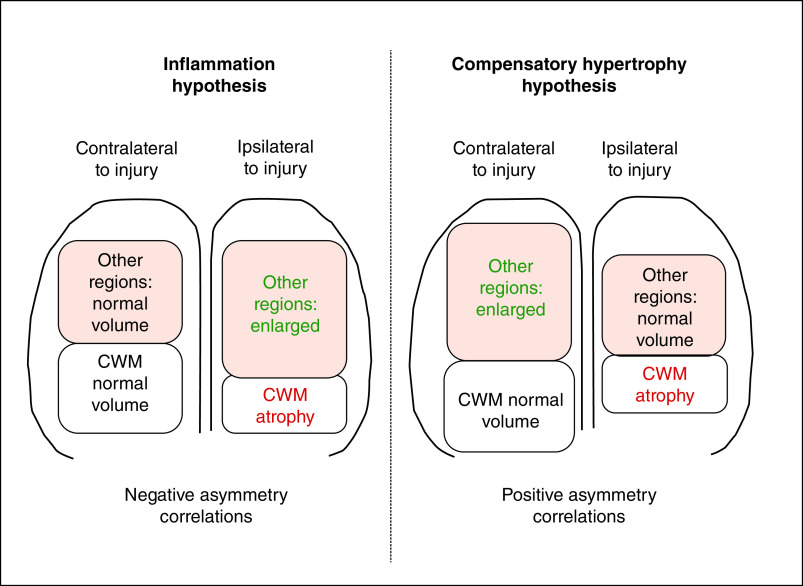
The two alternative hypotheses to explain abnormal brain enlargement, neuroinflammation versus compensatory hypertrophy, predicted different patterns of asymmetry correlations between cerebral white matter and enlarged brain regions within the patient group. CWM: Cerebral white matter.

In contrast, the neuroinflammation hypothesis would predict chronic ipsilateral CWM atrophy and abnormal enlargement of non-CWM regions (Figure 2, left side), for the following reasons: it is well known that TBI, including mild, moderate or severe TBI, is characterized chronically by CWM atrophy and, to our knowledge, not by CWM enlargement; and neuroinflammation, such as inflammation in general – which includes edema – is most likely to occur close to the site of forceful injury. Note that, in patients with TBI, inflammation does occur in CWM, even chronically; but when that occurs, it is associated with atrophy, possibly due to degradation of the tissue [15].

To present a crude analogy, if you hit your thumb with a hammer, the inflammation hypothesis would predict that you would have more enlargement of the injured thumb than the contralateral thumb, and the compensatory hypertrophy hypothesis would predict that, after the acute inflammation resolved, you would have a larger thumb contralateral to injury because you had been working it harder.

Although this case provided heuristic value for exploring mechanisms of injury, it did not allow the inference that similar processes occurred in other patients. But the previous reports did suggest an inferential method of testing the two competing hypotheses by using group data.

Assuming that injury on a given side of the brain would cause ipsilateral CWM atrophy, the two hypotheses made different predictions: the neuroinflammation hypothesis predicted that the injury would cause ipsilateral enlargement of non-CWM regions, and therefore CWM asymmetry would correlate negatively with the asymmetries of non-CWM regions (Figure 2, left side); and the compensatory hypertrophy hypothesis predicted that the injury would cause contralateral enlargement of non-CWM regions, and therefore CWM asymmetry would correlate positively with the asymmetries of non-CWM regions (Figure 2, right side).

Why would the two hypotheses predict this pattern of correlations? To explain this by way of example (Table 1), imagine that half of the patients had greater injury to the left side of the brain, leading to abnormal CWM asymmetry (L<R); we will call this subgroup ‘left-injured patients’. Next imagine that the other half of the patients had greater injury to the right side of the brain, leading to abnormal CWM asymmetry (R<L); we will call this subgroup ‘right-injured patients’.

**Table 1.** Detailed explanation of two competing hypotheses regarding brain volume and asymmetry in patients with chronic mild or moderate TBI using examples.

	Neuroinflammation hypothesis							Compensatory hypertrophy hypothesis						
	Left-injured TBI patients			Right-injured TBI patients			All TBI patients	Left-injured TBI patients			Right-injured TBI patients			All TBI patients
	**Vol**	**Asym**	**Direction of correlations between CWM and Non-CWM regions**	**Vol**	**Asym**	**Direction of correlations between CWM and Non-CWM regions**	**Direction of correlations between CWM and Non-CWM regions**	**Vol**	**Asym**	**Direction of correlations between CWM and Non-CWM regions**	**Vol**	**Asym**	**Direction of correlations between CWM and Non-CWM regions**	**Direction of correlations between CWM and Non-CWM regions**
CWM	L small	L<R	Negative	R small	R<L	Negative	Negative	L small	L<R	Positive (L<R = R>L)	R small	R<L	Positive (R<L = L>R)	Positive
Non-CWM regions	L large	L>R		R large	R>L			R large	R>L		L large	L>R		

This theoretical schema divides the group of TBI patients into two subgroups, those with left-sided cerebral injury (called ‘left-injured patients’) and those with right-sided cerebral injury (called right-injured patients). Both hypotheses predicted atrophy of CWM ipsilateral to the side of injury. In contrast, the two hypotheses predicted opposite effects on regions other than CWM, including cerebral cortical gray matter and subcortical regions (called ‘non-CMW regions’): the neuroinflammation hypothesis predicted ipsilateral enlargement, and the compensatory hypertrophy hypothesis predicted contralateral enlargement. These opposite effects on non-CWM regions led to opposite predictions for correlations between CWM asymmetry and non-CWM asymmetries within the TBI group: the neuroinflammation hypothesis predicated that the correlations would be negative, and the compensatory hypertrophy hypothesis predicted that they would be positive. See Introduction section for further details.

L<R: abnormal asymmetry with the left-sided brain region being smaller than expected based on the size of its contralateral counterpart; L>R: abnormal asymmetry with the left-sided brain region being larger than expected based on the size of its contralateral counterpart; R<L: abnormal asymmetry with the right-sided brain region being smaller than expected based on the size of its contralateral counterpart; R>L: abnormal asymmetry with the right-sided brain region being larger than expected based on the size of its contralateral counterpart; “L<R: R>L” indicates that, based on the definition of asymmetry for a given pair of brain regions, asymmetry (L<R) is equivalent to asymmetry (R>L); “R<L: L>R” indicates that asymmetry (L<R) is equivalent to asymmetry (R>L).

Asym: Asymmetry; CWM: Cerebral white matter; L: left; Non-CWM: Regions other than CWM, including cerebral cortical gray matter and subcortical regions; R: right; TBI: Traumatic brain injury; Vol: volume.

## Neuroinflammation hypothesis & the left-injured patient subgroup

Given this hypothetical schema, the neuroinflammation hypothesis predicted that the left-injured patients would have greater injury and inflammation near the site of forceful injury, that is, on the left side. This inflammation would lead to edema of left-sided non-CWM regions, causing enlargement and asymmetry (L>R). As noted above, this subgroup of patients would have asymmetry of the CWM in the opposite direction, i.e. (L<R). Therefore, correlations in this subgroup between CWM asymmetry (L<R) and non-CWM asymmetries (L>R) would be negative.

## Neuroinflammation hypothesis & the right-injured patient subgroup

Similarly, the neuroinflammation hypothesis predicted that the right-injured patients would have enlargement of non-CWM regions ipsilateral to the site of injury, i.e. right side, leading to asymmetry (R>L). This subgroup would have abnormal asymmetry of the CWM in the opposite direction, i.e. (R<L). Therefore, correlations in this subgroup between CWM asymmetry (R<L) and non-CWM asymmetries (R>L) also would be negative.

## Neuroinflammation hypothesis & the entire TBI group

In summary, the neuroinflammation hypothesis predicted that correlations between CWM asymmetry and non-CWM asymmetries would be negative for the left-injured patients and the right-injured patients. So overall the correlations would be negative for the entire group of TBI patients.

## Compensatory hypertrophy hypothesis & the left-injured patient subgroup

In contrast, the compensatory hypertrophy hypothesis predicted that the left-injured patients would have enlargement of regions on the side contralateral the injury–that is, the right side–because these regions were less injured and “worked harder” to compensate for the more-injured left-sided regions. This enlargement of the right-sided non-CWM regions would lead to asymmetry (R>L). As noted above, this subgroup would have abnormal asymmetry of the CWM (L<R). Based on the formula for calculating asymmetry, for a given pair of brain regions, asymmetry (R>L) is equivalent to asymmetry (L<R); in other words, they are two equivalent ways of looking at the same thing. Therefore, for this subgroup, the cerebral white asymmetry (L<R = R>L) would correlate positively with the non-CWM asymmetries (R>L = L<R).

## Compensatory hypertrophy hypothesis & the right-injured patient subgroup

Similarly, the compensatory hypertrophy hypothesis predicted that the right-injured patients would have enlargement of regions on the side contralateral injury–that is, the left side. This enlargement of the left-sided regions other than CWM would lead to abnormal asymmetry (L>R). As noted above, this subgroup of patients would have abnormal asymmetry of the CWM (R<L). Therefore, the cerebral white asymmetry (R<L = L>R) would correlate positively with the asymmetries of the other brain regions (L>R = R<L).

## Compensatory hypertrophy hypothesis & the entire TBI group

In summary, the compensatory hypertrophy hypothesis predicted that correlations between CWM asymmetry and non-CWM asymmetries would be positive for the left-injured patients and positive for the right-injured patients. So overall the correlations would be positive for the entire group of TBI patients.

The primary aims of this study were to determine: (1) whether patients with chronic mild or moderate TBI had abnormal brain asymmetry, and (2) whether asymmetry correlational analyses within the TBI patient group reflected the previously detected pattern of ipsilateral CWM atrophy correlating with contralateral non-CWM hypertrophy. Predicate aims were to test the reliability of the NeuroQuant^®^ brain volume meaurements, including test-retest reliability and reliability between NeuroQuant^®^ software versions 2.0 and 2.3.

## Materials & methods

### Participants

#### Patients

The sample of patients was the same as that studied and described in detail previously [11]. In brief, the patients were adult outpatients consecutively admitted to the Virginia Institute of Neuropsychiatry who had chronic mild or moderate TBI. All patients had symptoms that persisted for at least 6 months after injury, although a small minority had their brain MRI scan less than 6 months from injury (see below). Many of the patients had other disorders due to the TBI, including mood disorders, sleep disorders and posttraumatic stress disorder. Excluded were patients with other disorders that would affect brain volume measurement.

For the 50 patients who met the selection criteria, demographic characteristics were as follows: 26 men and 24 women; mean number of years of education was 14.3 (SD 3,0; range 10–21); mean age in years at the time of the injury was 46.7 (SD 12.5; range 16.9–80.2); mean age in years at the time of the MRI scan was 48.0 (SD 12.4; range 18.2–80.4); and mean interval between time of injury and time of MRI was 1.36 years (SD 1.14; range 0.11–5.68).

45 patients had mild TBI and 5 patients had moderate TBI. Causes of injury included the following (reported for all patients, only mild TBI patients, and only moderate TBI patients): motor vehicle accident (all patients *n* = 41; mild TBI *n* = 37; moderate TBI *n* = 4), train accident (all patients *n* = 4; mild TBI *n* = 4), hit in head with object (all patients *n* = 2; mild TBI *n* = 1; moderate TBI *n* = 1) fell down steps (all patients *n* = 1; mild TBI *n* = 1), mining accident (all patients *n* = 1; mild TBI *n* = 1) and motor vehicle versus pedestrian (all patients *n* = 1; mild TBI *n* = 1).

For the group of all 50 patients, the mean GCS score was 14.7, median 15, range 11–15. The mean duration of loss of consciousness (in minutes) was 3.1, median 0, range 0–30. The median duration of posttraumatic amnesia was 0.17 hours, range 0–264.00.

Regarding other neuropsychiatric symptoms due to the brain injury, in general, the sample of patients had a wide range of chronic symptoms, which caused them to seek treatment at a TBI specialty outpatient clinic. The mean score on the Glasgow Outcome Scale-Extended version (GOS-E) [23] was 5.4 (*SD* = 0.6), reflecting a patient sample in which about half of the patients were unable to return to gainful employment and the other half were gainfully employed but had significant problems functioning at work.

This study was approved by the Sterling Institutional Review Board and satisfied the requirements of the Code of Ethics of the World Medical Association (Declaration of Helsinki) for human research. Each patient signed an informed consent form to participate in research.

#### Normal control data

The normal control data came from the NeuroGage^®^ software produced by NeuroGage LLC (www.NeuroGage.com) (Midlothian, VA, USA). NeuroGage^®^ is based on NeuroQuant^®^ (for more information about NeuroGage^®^ and NeuroQuant^®^, see below).

As described in previous publications (for a summary, see [24]), the NeuroGage^®^ normal control group was a subgroup of the Alzheimer's Disease Neuroimaging Initiative (ADNI) [25–27]. Compared with the methods involving the previous NeuroGage^®^ software (version 1.0) [4,7,28] the current study used NeuroGage^®^ 2.0 software that was based on a similar but larger normal control group (N = 40 women and 40 men; total N = 80; mean age (in years) at time of MRI scan = 68.4, SD 3.2, median 70, range 60–72.

The NeuroGage^®^ normal controls were significantly older (mean = 68.4 years, *SD* = 3.2) than the patients (mean = 48.3, *SD* = 12.6) (t = -11.06, df = 53.1, p > 0.0001). The NeuroGage^®^ normal controls had significantly more years of education (mean = 16.4, *SD* = 2.7) than the patients (n = 50) (mean = 14.3, *SD* = 3.0) (t = -4.21, df = 128, p > 0.0001). (For a discussion of the effects of age and education on asymmetry, see the Statistical Analyses section below.)

### Brain imaging

#### Magnetic resonance imaging

Each patient had a 3.0 Tesla MRI of the brain performed at one of various radiology centers using the scanning protocol recommended for allowing later NeuroQuant^®^ analysis; this protocol is described in detail on the NeuroQuant^®^ website (http://www.cortechs.net/products/neuroquant.php). The MRI scanning protocol was the same as that described in our previous publication [11] including T1-weighted sequence, sagittal and 3D acquisition.

Normal control participants were scanned with either 1.5T or 3.0T scanners. The normal controls were selected from the Alzheimer's Disease Neuroimaging Initiative (ADNI) study, which initially used 1.5T scanners, and later used 3.0T scanners (http://adni.loni.usc.edu/methods/mri-tool/mri-analysis). NeuroQuant^®^ is FDA-cleared to be used on 1.5T or 3.0T scanners, indicating good reliablity between scanner strengths for the volume measurements (https://www.cortechslabs.com/resources/technical-information/recommended-scanner-settings).

All MRIs were interpreted by the attending radiologist using the traditional method of visual inspection. The vast majority were interpreted as having no volume abnormalities, consistent with our previous reports [4,29].

#### NeuroQuant^®^ software was used for brain volume measurement

MRI brain volume was measured using NeuroQuant^®^, a computer-automated, FDA-cleared method. Each participant was analyzed using NeuroQuant^®^ software, version 2.0 or 2.3. All the NeuroGage^®^ normal controls had NeuroQuant^®^ 2.0 Triage Brain Atrophy analyses. All the patients had NeuroQuant^®^ 2.3 Triage Brain Atrophy analyses. 30 brain regions overlapped between the 2.0 and 2.3 versions of the Triage analyses, but one of those (brainstem) did not have left and right regions analyzed separately and therefore could not be used for asymmetry analyses. The remaining 29 regions were used for the comparisons between the patients and NeuroGage^®^ normal controls. The volume results of these 2 software versions for these 29 overlapping regions were highly reliable with each other because the segmentation algorithm for NeuroQuant^®^ did not significantly change between 2.0 and 2.3; email communication on 01/31/19, Kelly Leyden, Product Manager, CorTechs Labs Inc. (We tested that reliability; see section 2.3.2 below). Therefore, both the 2.0 and 2.3 data were used for the analyses.

#### NeuroQuant^®^ automated brain MRI segmentation

The brain MRI data for each participant was uploaded to the NeuroQuant^®^ server, which processed and analyzed the brain imaging data. The output of the NeuroQuant^®^ computer-automated analysis included one or more reports which contained volumetric information, and a set of DICOM-formatted brain images which were segmented, with each region identified by a distinctive color.

The NeuroQuant^®^ segmented DICOM images were inspected for errors by one of the authors (DER), in order to ensure accurate identification of brain regions by the software. If a region was identified inaccurately by NeuroQuant^®^, it was omitted from the subsequent analyses. For the current study, there were 3770 regions identified (130 participants × 29 regions per participant); 1.0% of brain regions (36 errors per 3770 regions) were identified inaccurately; and therefore the associated volumes were omitted from any further analyses.

#### NeuroQuant^®^ brain volume analyses

All subjects were analyzed using the NeuroQuant^®^ Triage Brain Atrophy report (https://www.cortechslabs.com/products/neuroquant/tba), which included cortical gray matter, CWM, basal ganglia, infratentorial regions, and numerous cortical gray matter regions.

#### NeuroGage^®^ brain volume asymmetry analyses

NeuroGage^®^ 2.0 software was used to measure brain volume asymmetry and compare asymmetry between patients and normal control participants. NeuroGage^®^ is based on NeuroQuant^®^ and is designed to extend the utility of NeuroQuant^®^. Its reliability and validity have been supported by multiple peer-reviewed publications (for review, see [4]). For example, to analyze asymmetry, NeuroGage^®^ was used instead of NeuroQuant^®^ because NeuroQuant^®^ does not compare the asymmetry results to a normal control group, and therefore it is not possible to tell from NeuroQuant^®^ alone if the asymmetry results are normal or abnormal. NeuroGage^®^ uses its 80 normal controls to determine normal asymmetry and then can compare a group of patients (e.g. TBI patients in the current study) or single participants to the group of NeuroGage^®^ normal controls in order to determine if the asymmetries are normal or abnormal [4–6,24].

Regarding calculation of asymmetry, our methods have been described in detail previously [6]. In brief, asymmetry values were calculated using the same formula used by NeuroQuant^®^, that is: Asymmetry index = (Left_volume – Right_volume)/(Mean of Left_Volume and Right_Volume), expressed as a percentage. The asymmetry index calculated how much bigger was the left side versus the right, relative to the mean volume of the left and right sides. The formula used the mean volume of the left and right sides in the denominator as the best estimate of pre-injury volume of left or right side. The group of patients was compared with the NeuroGage^®^ normal control group.

### Statistical analyses

#### Inspection of distributions of data

Distributions of data were inspected for outliers and distributional characteristics. For data that were at least approximately normally distributed, parametrics statistics were used. For other data, nonparametric statistics were used.

#### Testing reliability

Test-retest reliability for the normal control participants (n = 80) was performed using our previously described methods [28,30]. Each normal control had repeat intra-scanner MRI testing around 1 year after the first MRI (interscan interval mean = 1.06 years; *SD* = 0.07 years) that was used for reliability testing. Although this period was longer than ideal for examining test-retest reliability, it was potentially useful insofar as it would provide a lower boundary on the test-retest reliability if performed under ideal conditions. That is, if the test-retest reliabilities were good, it would be safe to assume that they would be as good or better under ideal conditions.

In addition, because NeuroQuant^®^ software version 2.0 was used for the normal control participants, and NeuroQuant^®^ 2.3 was used for the patients, the reliability between the two software versions was tested for the 80 normal controls.

Reliabilities were calculated for each brain region using intraclass correlations coefficients (ICCs; 3, 1) using the terminology of Shrout and Fleiss [31] and were performed using SPSS version 25 (Model = 2-Way Mixed; Type = Absolute agreement; Confidence Interval = 95%). To interpret ICC values, the following guidelines were used: ICC <0.5 represented poor reliability, 0.5<ICC<0.75 represented moderate reliability, 0.75<ICC<0.9 represented good reliability, and ICC>0.9 represented excellent reliability [32].

#### Comparing asymmetry between groups

Given previous reports of effects of sex on brain asymmetry (for example, see [33–35]), the data were adjusted for the effects of sex as follows.

For each female participant, each brain region asymmetry was compared with that of the female normal control group (N = 40), and a Z score relative to the normal control data was calculated [where Z = (Individual_Patient's_asymmetry – Mean_of_Normals'_asymmetry)/(SD of Normals' asymmetry). A similar approach was used for the male participants, who were compared with the male normal control group (N = 40). The resultant sex-adjusted asymmetry Z scores were combined across the 2 sexes within each of the two groups (patients and normal controls) to maximize statistical power for comparing the two groups.

It was hypothesized that there would be no effects of age or education on asymmetry because, for each participant, both sides of the brain would be affected equally by both variables. This idea was tested by performing a series of Spearman's rho correlations on all asymmetry measures with respect to age and education in the normal control group (n = 80). The nonparametric Spearman rho was used instead of a parametric correlation because the age and education data for the normal controls were highly skewed.

There was a below-chance level number of significant correlations related to age (1/29 = 3.4% of correlations), confirming the hypothesis that age had no effects on asymmetry. Therefore, the asymmetry results were not adjusted for age based on the following reasons: (1) to our knowledge, there is no evidence that age affects brain volume asymmetry; (2) theoretically there would be no effect of age on asymmetry because both sides of the brain age at the same rate, so in essence, each subject's contralateral brain region serves as an internal control that adjusts for the effects of age; and (3) the analyses presented just above directly tested these ideas and found no evidence for effects of age above chance levels; and (4) although ideally it would have been preferable to test this hypothesis in normal controls age-matched to the patients, effects of age on brain volume generally are greater in older people than in younger people, making this a conservative limitation; that is, if age caused increased asymmetry, that would have made it more difficult to find significant differences between the younger patients and the older normal controls.

There was an above-chance level number of significant correlations related to education (3/29 = 10.3% of correlations). These included significant correlations between years of education and asymmetry in the primary sensory gyri (rho = 0.26, p = 0.02), posterior superior temporal sulcus regions (rho = -.34, p = 0.0024) and fusiform gyri (rho = -.24, p = 0.03). None of the P values survived the Bonferroni corrected alpha level of 0.05/29 = 0.0017. Furthermore, most of the rho values were small and none were large, indicating that effect sizes generally were small. These findings raised questions about whether education truly affected asymmetry. Nevertheless, in order to be conservative, no analyses involving asymmetry measures for these 3 brain regions were included in the comparisons between patients and normal controls.

All of the brain volume data were distributed at least approximately normally. Therefore, for the group comparisons involving asymmetry measures, *t*-tests were used. For each comparison, Levene's test was used to test for equality of variances between groups. If variances differed significantly, then an unequal variances (Welch's) *t*-test was used.

For group comparisons, Cohen's effect size d–defined as the difference in group means, divided by the pooled *SD*–were calculated and interpreted with reference to Cohen's scheme, where 0.2 is small, 0.5 is medium, and 0.8 is a large effect size difference between groups ([36] pp. 25–26).

#### Testing hypotheses with asymmetry correlations within the patient group

Pearson correlations were used to test the hypothesis that CWM asymmetry would correlate positively with asymmetries of 17 other brain regions that previously were found to be abnormally large (based on group comparisons) [11,12] and for which asymmetry data were available.

#### Statistical software

JMP Pro version 14.0.0 was used to perform all statistical analyses except the intraclass correlations, which were performed using SPSS version 25.

## Results

### Reliability

ICCs for test-retest analyses ranged from 0.78 to 1.00, indicating good to excellent reliability for all brain regions (Table 2).

**Table 2.** 1-year test–retest correlation coefficients for MRI brain volume regions for the NeuroGage^®^ normal controls (n = 80) based on NeuroQuant^®^ 2.0 analyses.

Brain region	Intraclass correlation coefficient		
	Left	Right	Mean
Whole brain parenchyma	0.99	0.99	0.99
Forebrain parenchyma	0.99	0.99	0.99
Cerebral white matter	0.98	0.98	0.98
Cerebral cortical gray matter	0.98	0.98	0.98
Subcortical nuclei + infratentorial regions	0.99	0.98	0.99
Ventricles:	0.99	1.00	1.00
– Superior lateral ventricles	0.99	0.99	0.99
– Third ventricle	0.97	0.98	0.98
– Inferior lateral ventricle	0.95	0.96	0.96
Cerebellum:	0.99	0.98	0.99
– Cerebellar white matter	0.85	0.87	0.86
– Cerebellar gray matter	0.98	0.98	0.98
Brainstem	0.99	0.98	0.99
Thalamus	0.96	0.95	0.96
Ventral diencephalon	0.91	0.90	0.91
Basal ganglia:	0.98	0.96	0.97
– Putamen	0.96	0.95	0.96
– Caudate	0.98	0.98	0.98
– Nucleus accumbens	0.94	0.94	0.94
– Pallidum	0.78	0.81	0.79
Cingulate:	0.93	0.94	0.93
– Anterior cingulate	0.91	0.94	0.92
– Posterior cingulate	0.9	0.85	0.87
– Isthmus cingulate	0.96	0.97	0.96
Frontal lobe:	0.97	0.97	0.97
– Superior frontal	0.95	0.95	0.95
– Middle frontal	0.90	0.92	0.91
– Inferior frontal	0.94	0.92	0.93
– Lateral orbitofrontal	0.94	0.91	0.93
– Medial orbitofrontal	0.93	0.96	0.94
– Paracentral	0.90	0.86	0.88
– Primary motor	0.91	0.92	0.92
Parietal lobe:	0.96	0.96	0.96
– Primary sensory	0.93	0.90	0.92
– Medial parietal	0.94	0.96	0.95
– Superior parietal	0.92	0.87	0.9
– Inferior parietal	0.90	0.92	0.91
– Supramarginal	0.92	0.90	0.91
Occipital lobe:	0.96	0.95	0.96
– Medial occipital	0.95	0.93	0.94
– Lateral occipital	0.92	0.92	0.92
Temporal lobe:	0.97	0.96	0.97
– Transverse temporal + superior temporal	0.96	0.97	0.97
– Posterior superior temporal sulcus	0.94	0.92	0.93
– Middle temporal	0.95	0.95	0.95
– Inferior temporal	0.90	0.90	0.90
– Fusiform	0.92	0.93	0.93
– Parahippocampal	0.96	0.94	0.95
– Entorhinal cortex	0.94	0.88	0.91
– Temporal pole	0.86	0.88	0.87
Amygdala	0.95	0.92	0.94
Hippocampus	0.96	0.94	0.95

Intraclass correlation coefficients ranged from good to excellent.

ICCs comparing NeuroQuant^®^ 2.0 versus 2.3 software ranged from 0.95 to 1.00 (Table 3), indicating excellent reliability for all brain regions.

**Table 3.** NeuroQuant^®^ 2.0 versus NeuroQuant^®^ 2.3 intraclass correlation coefficients for MRI brain volume regions for the NeuroGage^®^ normal controls (n = 80) based on time 1 data.

Brain region	Intraclass correlation coefficient		
	Left	Right	Mean
Whole brain parenchyma	1.00	1.00	1.00
Forebrain parenchyma	1.00	1.00	1.00
Cerebral white matter	1.00	1.00	1.00
Cerebral cortical gray matter	1.00	1.00	1.00
Subcortical nuclei + infratentorial regions	1.00	0.99	1.00
Ventricles:	1.00	1.00	1.00
– Superior lateral ventricles	1.00	1.00	1.00
– Third ventricle	0.99	1.00	1.00
– Inferior lateral ventricle	0.99	0.99	0.99
Cerebellum:	1.00	1.00	1.00
– Cerebellar white matter	0.97	0.95	0.96
– Cerebellar gray matter	1.00	1.00	1.00
Brainstem	1.00	1.00	1.00
Thalamus	1.00	1.00	1.00
Ventral diencephalon	0.97	0.97	0.97
Basal ganglia:	1.00	1.00	1.00
– Putamen	1.00	1.00	1.00
– Caudate	1.00	1.00	1.00
– Nucleus accumbens	0.99	0.97	0.98
– Pallidum	0.98	0.96	0.97
Cingulate:	0.99	0.99	0.99
– Anterior cingulate	0.99	1.00	1.00
– Posterior cingulate	0.98	0.98	0.98
– Isthmus cingulate	0.99	0.99	0.99
Frontal lobe:	1.00	1.00	1.00
– Superior frontal	1.00	1.00	1.00
– Middle frontal	1.00	1.00	1.00
– Inferior frontal	1.00	1.00	1.00
– Lateral orbitofrontal	1.00	0.99	1.00
– Medial orbitofrontal	0.99	1.00	1.00
– Paracentral	0.99	0.99	0.99
– Primary motor	1.00	1.00	1.00
Parietal lobe:	1.00	1.00	1.00
– Primary sensory	1.00	0.99	1.00
– Medial parietal	1.00	1.00	1.00
– Superior parietal	1.00	1.00	1.00
– Inferior parietal	1.00	1.00	1.00
– Supramarginal	0.99	1.00	1.00
Occipital lobe:	1.00	1.00	1.00
– Medial occipital	1.00	1.00	1.00
– Lateral occipital	1.00	1.00	1.00
Temporal lobe:	1.00	1.00	1.00
– Transverse temporal + superior temporal	1.00	1.00	1.00
– Posterior superior temporal sulcus	0.99	0.99	0.99
– Middle temporal	1.00	1.00	1.00
– Inferior temporal	1.00	1.00	1.00
– Fusiform	0.99	1.00	1.00
– Parahippocampal	0.99	0.98	0.99
– Entorhinal cortex	1.00	0.99	1.00
– Temporal pole	0.99	0.99	0.99
Amygdala	0.99	0.98	0.99
Hippocampus	0.99	0.98	0.99
All intraclass correlation coefficients were ≥0.95, indicating excellent reliability.			

### Comparing asymmetry between groups

The groups of patients and NeuroQuant^®^ normal controls were compared with respect to MRI brain volume asymmetries (Table 4). The patients had more pairs of regions with abnormal asymmetry (7 of 26 pairs = 26.9%) than was expected by chance alone (5% with leftward asymmetry + 5% with rightward asymmetry = 10% total; 10% of 26 pairs = 2.6 pairs expected by chance alone).

**Table 4.** Comparisons of brain volume asymmetries (Z scores) between patients and normal controls showed that the patients had several regions of abnormal asymmetry.

Brain region	Group	Mean	SD	t	df	Sig. (2-tailed)	Effect size d
Cerebral white matter	Patient	-0.15	1.38	-0.73	126	0.47	-0.1
	Normal	0.00	1.00				
Cortical gray matter	Patient	-0.38	1.02	-2.05	126	**0.04** ^†^	**-0.4**
	Normal	0.00	1.00				
Superior lateral ventricles	Patient	0.05	1.56	0.18	75	0.85	0.0
	Normal	0.00	1.00				
Cerebellar white matter	Patient	0.09	1.08	0.49	128	0.62	0.1
	Normal	0.00	1.00				
Cerebellar gray matter	Patient	0.42	0.93	2.39	128	**0.02** ^†^	**0.4**
	Normal	0.00	1.00				
Thalamus	Patient	-0.18	1.09	-0.98	128	0.33	-0.2
	Normal	0.00	1.00				
Ventral diencephalon	Patient	-0.36	0.98	-1.96	127	0.05	-0.4
	Normal	-0.01	1.00				
Putamen	Patient	0.05	1.01	0.28	128	0.78	0.1
	Normal	0.00	1.00				
Caudate	Patient	-0.07	1.10	-0.37	128	0.71	-0.1
	Normal	0.00	1.00				
Nucleus accumbens	Patient	-0.30	1.19	-1.52	128	0.13	-0.3
	Normal	0.00	1.00				
Pallidum	Patient	0.53	0.94	2.98	128	**0.004** ^†^	**0.5**
	Normal	0.00	1.00				
Superior frontal gyrus	Patient	-0.55	1.26	-2.69	125	**0.008** ^†^	**-0.5**
	Normal	0.00	1.00				
Lateral orbitofrontal gyrus	Patient	0.01	0.87	0.05	126	0.96	0.0
	Normal	0.00	1.00				
Primary motor region	Patient	0.33	1.01	1.78	125	0.08	0.3
	Normal	0.00	1.00				
Primary sensory region	Patient	-0.14	1.23	-0.72	126	0.47	-0.1
	Normal	0.00	1.00				
Medial parietal	Patient	0.82	1.42	3.85	126	**0.0002** ^†^	**0.7**
	Normal	0.00	1.00				
Superior parietal	Patient	-0.08	1.03	-0.48	126	0.63	-0.1
	Normal	0.01	0.99				
Inferior parietal	Patient	-0.01	1.10	-0.03	126	0.98	0.0
	Normal	0.00	1.00				
Supramarginal	Patient	0.15	0.92	0.83	126	0.41	0.2
	Normal	0.00	1.00				
Medial occipital	Patient	0.17	0.84	0.99	126	0.33	0.2
	Normal	0.00	1.00				
Lateral occipital	Patient	-0.05	1.02	-0.27	126	0.79	0.0
	Normal	0.00	1.00				
Transverse temporal + superior temporal	Patient	-0.36	1.27	-1.80	126	0.07	-0.3
	Normal	0.00	1.00				
Posterior superior temporal sulcus	Patient	-0.67	0.88	-3.93	126	**0.0001** ^†^	**-0.7**
	Normal	0.03	1.02				
Middle temporal	Patient	0.07	0.88	0.39	126	0.70	0.1
	Normal	0.00	1.00				
Inferior temporal	Patient	0.05	1.02	0.28	126	0.78	0.1
	Normal	0.00	1.00				
Fusiform	Patient	-0.17	0.79	-1.02	126	0.31	-0.2
	Normal	0.00	1.00				
Temporal pole	Patient	-0.38	0.85	-2.37	126	**0.02** ^†^	**-0.4**
	Normal	0.00	1.00				
Amygdala	Patient	0.34	0.82	1.99	128	**0.049** ^†^	**0.4**
	Normal	0.00	1.00				
Hippocampus	Patient	0.46	1.08	2.46	126	**0.02** ^†^	**0.4**
	Normal	0.00	1.00				

† Comparisons associated with p < 0.05.

It would be possible that, for a given brain region, a significant subgroup of patients would have abnormal asymmetry (e.g. L<R) but another subgroup would have abnormal asymmetry in the opposite direction (R<L). In that case, the means might not differ between the groups of patients and normal controls, despite the abnormalities in the patient subgroups. This possiblity could be tested by comparing variances between the groups of patients and normal controls, which would be hypothesized to be larger in the patient group. Accordingly, all pairs of regions were compared between groups for inequality of variances using Levene's test. Only 1 pair of regions (the left and right superior lateral ventricles) had a variance for the patients (*SD* = 1.56) that was significantly larger than that of the NeuroGage^®^ normal controls (*SD* = 1.00) (Levene's test: *F* = 11.82, *df* = 1,127, p > 0.001). The patient group had fewer pairs of regions with abnormal variance (1 of 26 pairs = 3.8%) than was expected by chance alone (5%), raising concern about whether the single abnormal finding was due to chance alone. In order to address this concern, the alpha level was adjusted for multiple comparisons using the Bonferroni adjustment (adjusted alpha level = 0.05/29 tests = 0.002) and the p value associated with the superior lateral ventricles (p > 0.001) survived the adjustment.

### Testing hypotheses with asymmetry correlations within the patient group

Examination of Pearson correlations (Table 5) between CWM asymmetry and the asymmetries of non-CWM regions (i.e. 17 brain regions other than CWM that previously were found to be abnormally large [11,12]) and for which asymmetry data were available showed the following significant correlations: cortical gray matter (*R* = 0.29, p = 0.05), thalamus (*R* = 0.33, p = 0.02), caudate (*R* = 0.61, p > 0.001), primary motor (*R* = 0.29, p = 0.05) and hippocampus (*R* = 0.31, p = 0.03). There were more statistically significant correlations (5) than were expected by chance alone (0.9 = 5% × 17 correlations).

**Table 5.** Correlations between cerebral white matter asymmetry and asymmetries of other brain regions showed multiple positive correlations.

Variable	R	p-value
Cerebral cortical gray matter	0.29	0.05^†^
Cerebellar white matter	0.16	0.27
Thalamus	0.33	0.02^†^
Ventral diencephalon	-0.08	0.58
Caudate	0.61	<0.001^†^
Nucleus accumbens	0.19	0.19
Primary motor	0.29	0.05^†^
Primary sensory	0.05	0.75
Medial parietal	0.05	0.74
Superior parietal	0.08	0.58
Inferior parietal	0.12	0.41
Medial occipital	0.15	0.31
Posterior superior temporal sulcus	0.10	0.51
Middle temporal	0.23	0.11
Fusiform	0.01	0.97
Amydgala	0.05	0.71
Hippocampus	0.31	0.03^†^

† Comparisons associated with p < 0.05.

## Discussion

### Abnormal brain asymmetry

This study was, to our knowledge, the first to perform group comparisons of brain asymmetries between normal controls and patients with chronic mild or moderate TBI. The patients had abnormal asymmetry of the cerebellar gray matter, amygdala, hippocampus, and several cortical gray matter regions. Also, the patients had variance in the distribution of asymmetry of the superior lateral ventricles that was significantly greater than that of the normal controls, indicating that a significant subgroup of patients had abnormal asymmetry (L<R), while another subgroup of patients had the opposite finding (R<L). These findings confirmed the hypothesis that patients with chronic mild or moderate TBI have multiple abnormal brain volume asymmetries.

### Testing hypotheses with asymmetry correlations within the patient group

**Figure 3.**
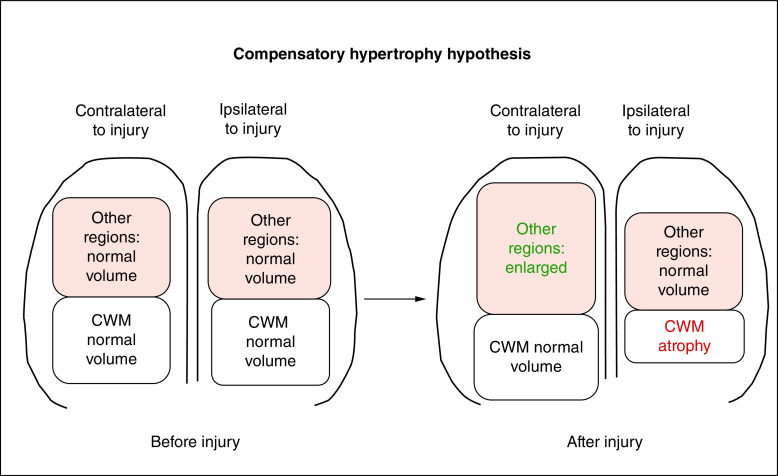
Summary diagram of the compensatory hypertrophy hypothesis, which posits that in patients with chronic mild or moderate traumatic brain injury, acute injury causes ipsilateral atrophy of cerebral white matter and eventually contralateral enlargement of less-injured regions. CWM: Cerebral white matter.

There were multiple positive correlations between CWM asymmetry and the non-CWM asymmetries. All the correlations were positive, supporting the hypothesis of compensatory hypertrophy (Table 1 & Figures 2 & 3).

### Limitations

Limitations of the current study included that this was not a longitudinal study: Volumes (including symmetries or asymmetries) were estimated to be normal just before injury, but they were not measured before injury. Abnormal volumes or asymmetries could have been present before injury, although the screening criteria used minimized that possibility. Future longitudinal studies will be needed to definitively address that limitation.

Our patient sample included 45 patients with chronic mild TBI and 5 patients with chronic moderate TBI. Accordingly, the results are more likely to apply to populations with similar characteristics, that is, patients mostly with chronic mild TBI, and not patients only with chronic moderate TBI or more severe TBI.

The current study examined patients with mild or moderate TBI who had symptoms that persisted for months to years after the injury and sought treatment at a TBI specialty clinic. Therefore, these results may not apply to other TBI patients, for example, patients with mild TBI who have complete resolution of symptoms within hours to days.

Finally, our study did not examine information related to side of brain injury in each patient. First, note that our sample of TBI patients included only those with closed head injury (i.e. patients who did not have penetrating skull injuries or skull fractures). In these types of patients, both sides of the brain usually are affected somewhat, due to coup-contrecoup forces, twisting forces, and other forces acting on brain tissues of different densities. Nevertheless, we still could consider patients with closed head injury who had greater injury to one side of the brain than the other. How would this be identified or measured? In some patients, it might be possible to identify the side of greater injury, for example, if there was a clear unilateral injury to the head, or if they had unilateral intraparenchymal bleeding. Unfortunately, using such criteria, side of greater injury could be identified for only a few patients in the current study, which would not have allowed the types of group-based analyses that were performed. In the future, it would be of interest to study patients for whom side of injury could be determined definitively a priori. Also, based on the findings of the current study, future investigators might consider designs based on using asymmetries (e.g., CWM or ventricles) as independent variables, for example, comparing a group patients with CWM asymmetry (L<R) to a group of patients with CWM asymmetry (R<L).

## Conclusion

The current study found that patients with chronic mild or moderate TBI had several regions of abnormal asymmetry, and within the patient group, CWM asymmetry correlated positively with the asymmetries in several other brain regions. Taken together, these findings supported the hypothesis of compensatory hypertrophy, that is, that the acute injury caused ipsilateral white matter atrophy, leading eventually to compensatory hypertrophy of contralateral non-CWM regions. The findings did not support the competing hypothesis of ipsilateral edema and enlargement of non-CWM regions.

These conclusions should be tested further with longitudinally-designed studies. But for practical reasons, often it is difficult to perform longitudinal studies; for example, it is rare to have brain MRIs performed before injury that would allow later longitudinal volume analyses, e.g., for measuring change from before to after injury. And given the fact that cross-sectional asymmetry analyses may be sensitive indicators of previous longitudinal changes, such analyses may be helpful for understanding the nature of brain injury in these patients.

The findings of the current study raise interesting questions about how they might be related to pathophysiology, metabolism, function, symptoms or course of illness. One question is of particular interest: if the pattern of asymmetries was due to compensatory hypertrophy, would that be helpful or harmful to patients? We speculate that it would be helpful in the short run because it involved compensation for functional or pathophysiological deficits, but in the long run it would be harmful because it would tax the functional or physiological capabilities of the non-CWM regions. (A crude analogy would be if a person lost the use of one arm and began using the remaining arm more to compensate; in the short run, the compensatory response would lead to a more highly functional arm, but in the long run, the arm would become weaker due to overuse injury.) If it is true that compensatory hypertrophy was helpful in the short run but harmful in the long run, it is possible that earlier treatments or interventions would prevent longer-term overuse injury to the non-CWM regions. Further research would be needed to explore these questions.

## Future perspective

The authors predict that volumetric brain imaging in patients with chronic mild or moderate traumatic brain injury (TBI) will become more important in the next 5 to 10 years. Brain MRIs will be performed closer to the time of injury (including the day of injury) and more frequently (e.g. on the day of injury, 1 week later, 1 month later, 3 months later etc). These improvement will allow better longitudinal analyses of volume change. Also artificial intelligence, including machine learning, will be used increasingly to distinguish patterns of brain volume abnormalities that differ between patient groups and normal controls. All these advancements will help researchers and clinicians better understand the pathophysiology of TBI, more accurately diagnose it, provide more objective evidence of brain injury–which for example would be important for forensic purposes–and better prognose and treat patients.

**Summary points**

- There is a substantial amount of literature reporting that patients with severe traumatic brain injury have brain atrophy and asymmetry, but much less is known about brain volume abnormalities in patients suffering from chronic effects of mild or moderate traumatic brain injury. Recent studies reported that patients with chronic mild or moderate traumatic brain injury had some regions of brain atrophy (including cerebral white matter [CVM) but, surprisingly, even more regions of abnormal brain enlargement (including cerebral cortical gray matter and subcortical regions) (herein called “non-CWM regions”).
- Consistent with this pattern, a recent case report described a patient who had day-of-injury left cerebral hemorrhage, followed 1.8 years later by left CWM atrophy but contralateral (i.e. right) non-CWM regions. These findings suggested the hypothesis that ipsilateral injury and CWM atrophy caused the eventual development of contralateral compensatory hypertrophy and asymmetry of non-CWM regions.
- The purpose of the current study was to use group data to determine whether patients with chronic mild or moderate traumatic brain injury have abnormal brain asymmetry and, if so, whether any asymmetry might confirm the previously detected pattern of ipsilateral white matter atrophy correlating with contralateral non-CWM enlargement and asymmetry.
- 50 patients with mild or moderate traumatic brain injury were compared with 80 normal control participants with respect to MRI brain volume asymmetry. Within the patient group, correlational analyses using the asymmetry data were used to test the hypothesis suggested by the case report, specifically, that white matter asymmetry would correlate positively, not negatively, with non-CWM asymmetries.
- The group of patients had multiple regions of abnormal asymmetry. Consistent with the hypothesis of compensatory hypertrophy, there were multiple positive correlations within the patient group based on the asymmetry data.
- These findings supported the interpretation that acute injury to ipsilateral CWM regions caused atrophy, leading eventually to abnormal enlargement and asymmetry of contralateral non-CWM regions due to compensatory hypertrophy.
